# ATF5 deficiency causes abnormal cortical development

**DOI:** 10.1038/s41598-021-86442-5

**Published:** 2021-03-31

**Authors:** Mariko Umemura, Yasuyuki Kaneko, Ryoko Tanabe, Yuji Takahashi

**Affiliations:** Laboratory of Environmental Molecular Physiology, School of Life Sciences, Tokyo University of Pharmacy and Life Sciences, Hachioji, Tokyo 192-0392 Japan

**Keywords:** Neural progenitors, Neuronal development, Autism spectrum disorders

## Abstract

Activating transcription factor 5 (ATF5) is a member of the cAMP response element binding protein (CREB)/ATF family of basic leucine zipper transcription factors. We previously reported that ATF5-deficient (ATF5^−/−^) mice exhibited behavioural abnormalities, including abnormal social interactions, reduced behavioural flexibility, increased anxiety-like behaviours, and hyperactivity in novel environments. ATF5^−/−^ mice may therefore be a useful animal model for psychiatric disorders. ATF5 is highly expressed in the ventricular zone and subventricular zone during cortical development, but its physiological role in higher-order brain structures remains unknown. To investigate the cause of abnormal behaviours exhibited by ATF5^−/−^ mice, we analysed the embryonic cerebral cortex of ATF5^−/−^ mice. The ATF5^−/−^ embryonic cerebral cortex was slightly thinner and had reduced numbers of radial glial cells and neural progenitor cells, compared to a wild-type cerebral cortex. ATF5 deficiency also affected the basal processes of radial glial cells, which serve as a scaffold for radial migration during cortical development. Further, the radial migration of cortical upper layer neurons was impaired in ATF5^−/−^ mice. These results suggest that ATF5 deficiency affects cortical development and radial migration, which may partly contribute to the observed abnormal behaviours.

## Introduction

Activating transcription factor 5 (ATF5) is a member of the cAMP response element binding protein (CREB)/ATF family of basic leucine zipper transcription factors^1^. Our group and others have reported that ATF5 is a stress responsive transcription factor under conditions such as endoplasmic reticulum (ER) stress and oxidative stress^2–4^. In the embryonic brain, ATF5 is highly expressed in neural progenitor cells in the ventricular zone (VZ) and subventricular zone (SVZ) where newly born neurons are generated^5–7^. In the adult brain, ATF5 is expressed in neurons in the cerebral cortex, striatum, hippocampus, and cerebellum^7^. ATF5 is essential for the proliferation and differentiation of progenitor cells and plays a neuroprotective role against ER stress^5,7^. However, the functions of ATF5 in neurogenesis and neuronal maturation remain unknown. ATF5-deficient (ATF5^−/−^) mice exhibit behavioural abnormalities, including reduced social interaction and behavioural flexibility, increased anxiety-like behaviours, and hyperactivity in novel environments^8^. This report suggests a potential link between ATF5 and the behavioural etiology of psychiatric disorders^8^. Therefore, ATF5^−/−^ mice may be considered a useful model for studying the pathology of psychiatric disorders. Elucidation of the pathogenesis of psychiatric disorders is a critical unmet need. In this regard, investigations of the mechanisms underpinning the behavioural abnormalities exhibited by ATF5^−/−^ mice may shed light on human psychiatric disorders.

It has been reported that autistic behavioural abnormalities, including reduced social interaction, are related to altered cortical development^9–12^. The cerebral cortex is the largest neural tissue in the brain. This tissue is located in the most superficial region of each cerebral hemisphere and is involved in cognition, emotional, and sensorimotor functions. The mammalian cerebral cortical structure comprises six functionally distinct layers. Each cortical layer contains a characteristic neuronal subset, and the neurons in each layer project to different brain regions. During development, newly born neural progenitors are generated by proliferation of radial glial cells and migrate radially toward the pial surface from the VZ^13–16^. In the mouse cerebral cortex, the earliest cortical neurons appear during embryonic day 10 (E10)–11 and become layer V–VI neurons^17,18^. Subsequent neurons, which become layer II–IV pyramidal cells via intermediate progenitor cells (IPC) appear at E13–16 and migrate past earlier born neurons to reach the more superficial layers in the cortical plate, resulting in the generation of an ‘inside-out’ pattern of cerebral cortical lamination.

Cortical neurons arise from radial glial cells, which are the neural progenitor cells that line the VZ^14,16^. A radial glial cell is a radial bipolar-shaped cell with an apical process extending to the ventricular surface and a basal process extending to the pial membrane which acts as a scaffold for the radial migration of neurons. During the early stages of cortical development, radial glial progenitors divide symmetrically to generate radial glial cells. Subsequently, radial glial cells divide asymmetrically to generate neural progenitor cells and IPCs. After the neurogenic phase (approximately E18 in mice), radial glial cells translocate toward the pial surface and differentiate into astrocytes. Radial glial cells also provide a scaffold for the radial migration of newly born neurons during locomotion. Defects in radial migration can lead to brain malformations such as lissencephaly and microcephaly, as well as psychiatric disorders such as bipolar disorder, epilepsy, autism spectrum disorder (ASD), and schizophrenia^12,13,19^.

Here, we demonstrate abnormal cerebral cortical development in ATF5^−/−^ mice. Compared to wild-type mice, ATF5^−/−^ mice had a thinner cerebral cortex and exhibited defective radial glial cell maintenance and an abnormal cell morphology. Adult ATF5^−/−^ mice had an irregular cerebral cortical laminar structure. Collectively, our findings suggest that ATF5 regulates cerebral cortical development.

## Results

### ATF5 is essential for cortical development

**Figure 1:**
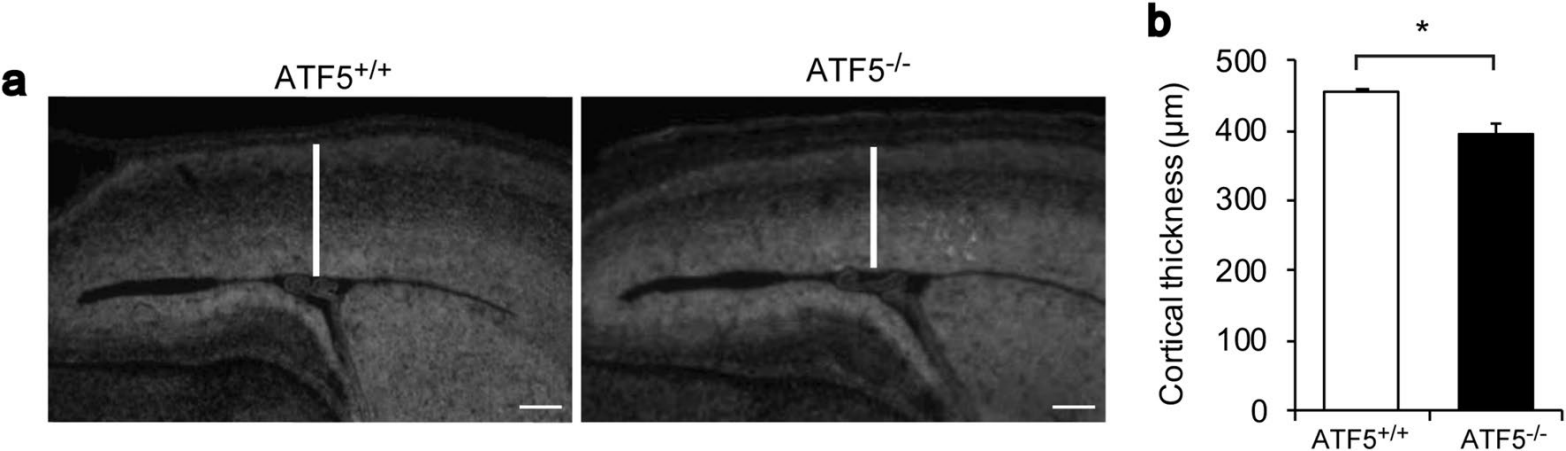
The reduced cortical thickness in the developing cortices of ATF5^−/−^ mice. (**a**) Cortical sections obtained on embryonic day 15 (E15) were stained with Hoechst 33342 to label the nuclei prior to an analysis of cortical thickness (solid line). (**b**) Quantification of the cortical thickness. Data are presented as mean ± S.E.M. (*n* = 3 mice per group, *p < 0.05; Student’s t-test). Scale bars represent 100 µm.

During cortical development, ATF5 is expressed in the VZ and SVZ, where newly born neurons are generated^7^. We hypothesised that ATF5-deficient mice would have impaired development of the cerebral cortex. On E15 in ATF5^−/−^ mice, cortical thickness was reduced compared with that in wild-type ATF5^+/+^ mice (Fig. 1), although the brain generally appeared normal except for the olfactory bulb. We previously reported that neonatal ATF5^−/−^ mice exhibited a normal whole-brain weight and reduced olfactory bulb size^20^. These results predicted defects in embryonic cortical development in ATF5^−/−^ mice and suggested that ATF5 deficiency would affect cortical development in the embryonic phase.

### ATF5 regulated the number of radial glial cells and IPCs in the developing cortex

**Figure 2:**
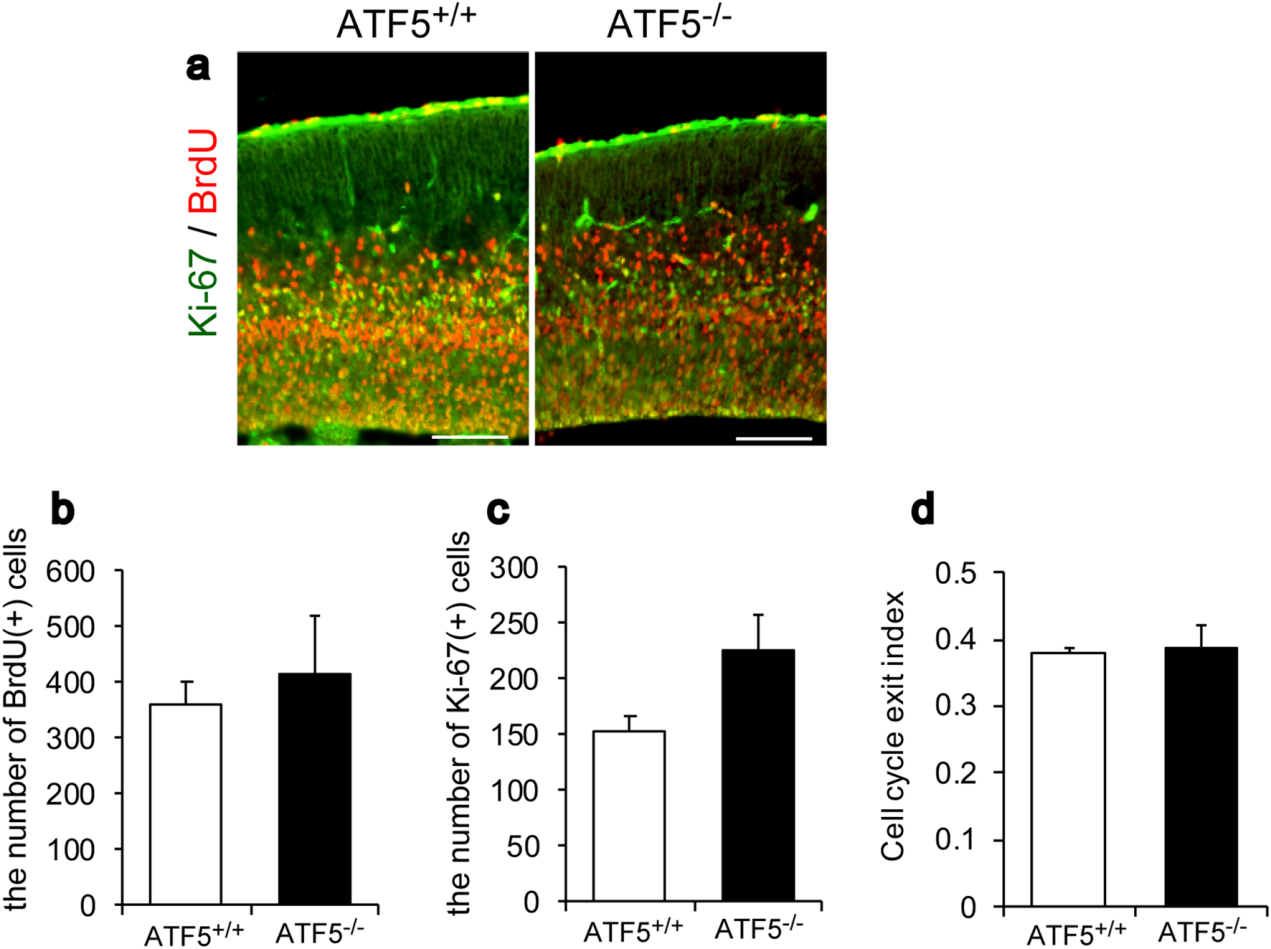
Analysis of proliferating cells in the developing cortices of ATF5^−/−^ mice. (**a**) Immunostaining for Ki-67 and BrdU (markers of proliferating cells) on embryonic day 15 (E15). Pregnant mice were injected intraperitoneally with BrdU (200 mg/kg body weight) on E14 and dissected after 24 h. (**b**–**d**) Quantification of the numbers of BrdU-positive cells (**b**) and Ki-67-positive cells (**c**), and the cell cycle exit index (**d**, BrdU( +) Ki-67( −) / all BrdU ( +) cells) on E15. Data are presented as mean ± S.E.M. (*n* = 3 mice per group). Scale bars represent 50 µm.

**Figure 3:**
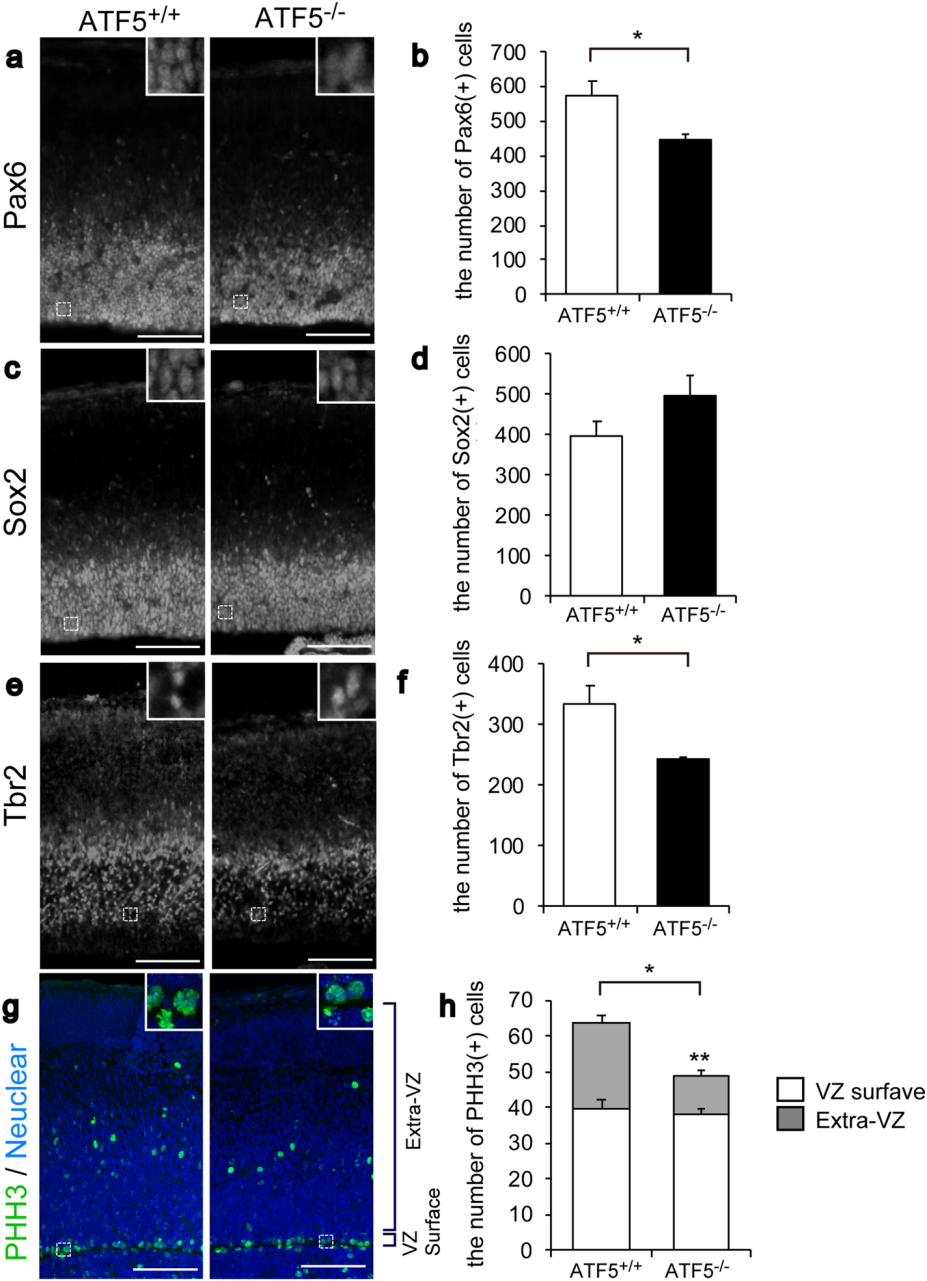
Reduced numbers of radial glial cells and intermediate cells in the developing cortices of ATF5^−/−^ mice. (**a**–**d**) Immunostaining for the radial glial cell markers Pax6 (**a**) and Sox2 (**c**) in cortical sections on embryonic day 15 (E15). (**e**,**f**) Immunostaining for Tbr2, an intermediate cell marker, in cortical sections on E15. (**b**,**d**,**f**) Quantification of the numbers of Pax6-positive cells (**b**), Sox2-positive cells (**d**), and Tbr2-positive cells (**f**) in the cortex on E15. (**g**,**h**) Immunostaining for PHH3 (**g**), a mitotic cell marker, in cortical sections on E15. (**f**) Quantification of the number of PHH3-poritive cells in the VZ surface region and in the extra-VZ regions in the cortex on E15. Each inset shows an enlarged view of the dotted line box. Data are presented as mean ± S.E.M. (*n* = 3 mice per group, *p < 0.05, **p < 0.01; Student’s t-test). Scale bars represent 100 µm.

To investigate the role of ATF5 during cortical development, cerebral cortical sections of ATF5^+/+^ and ATF5^−/−^ embryonic mice were immunostained using antibodies specific for neural developmental markers. Bromodeoxyuridine (BrdU) labelling coupled with immunostaining for the proliferation marker Ki-67 revealed no differences in the numbers of BrdU- and Ki-67-positive cells between ATF5^+/+^ and ATF5^−/−^ mice (Fig. 2a–c). The cell cycle exit indices^21^ of the two genotypes were not different (Fig. 2d). However, immunostaining for Pax6, a radial glial cell marker, revealed lower Pax6-positive cell numbers in ATF5^−/−^ E15 mice than in ATF5^+/+^ mice (Fig. 3a,b), although the number of Sox2-positive cells, an apical progenitor cell marker, was not significantly different (Fig. 3c,d) between ATF5^+/+^ and ATF5^−/−^ mice.

Radial glial cells and apical progenitor cells divide and give rise to neuronal progenitor cells known as IPCs. Immunostaining for Tbr2, an IPC marker, revealed lower Tbr2-positive cell numbers in ATF5^−/−^ mice (Fig. 3e,f). Immunostaining for phosphorylated histone H3 (PHH3), a mitotic cell marker, revealed PHH3-positive mitotic cells both at the VZ surface and distal to the VZ surface (extra-VZ) (Fig. 3g,h). Mitotic cells at the VZ surface are characteristic of radial glial cells, while those distal to the VZ surface are characteristic of IPCs^22,23^. The number of PHH3-positive mitotic cells distal to the VZ (but not those at the VZ surface) was significantly lower in ATF5^−/−^ mice compared to ATF5^+/+^ mice (Fig. 3g,h), consistent with the reduced IPC numbers in the developing cortex of ATF5^−/−^ mice. These results suggested that the populations of radial glial cells and IPCs were reduced in ATF5^−/−^ mice during cortical development, and ATF5 was sufficient for maintaining the numbers of radial glial cells and IPCs.

### ATF5 deficiency affected immature neuron production

**Figure 4:**
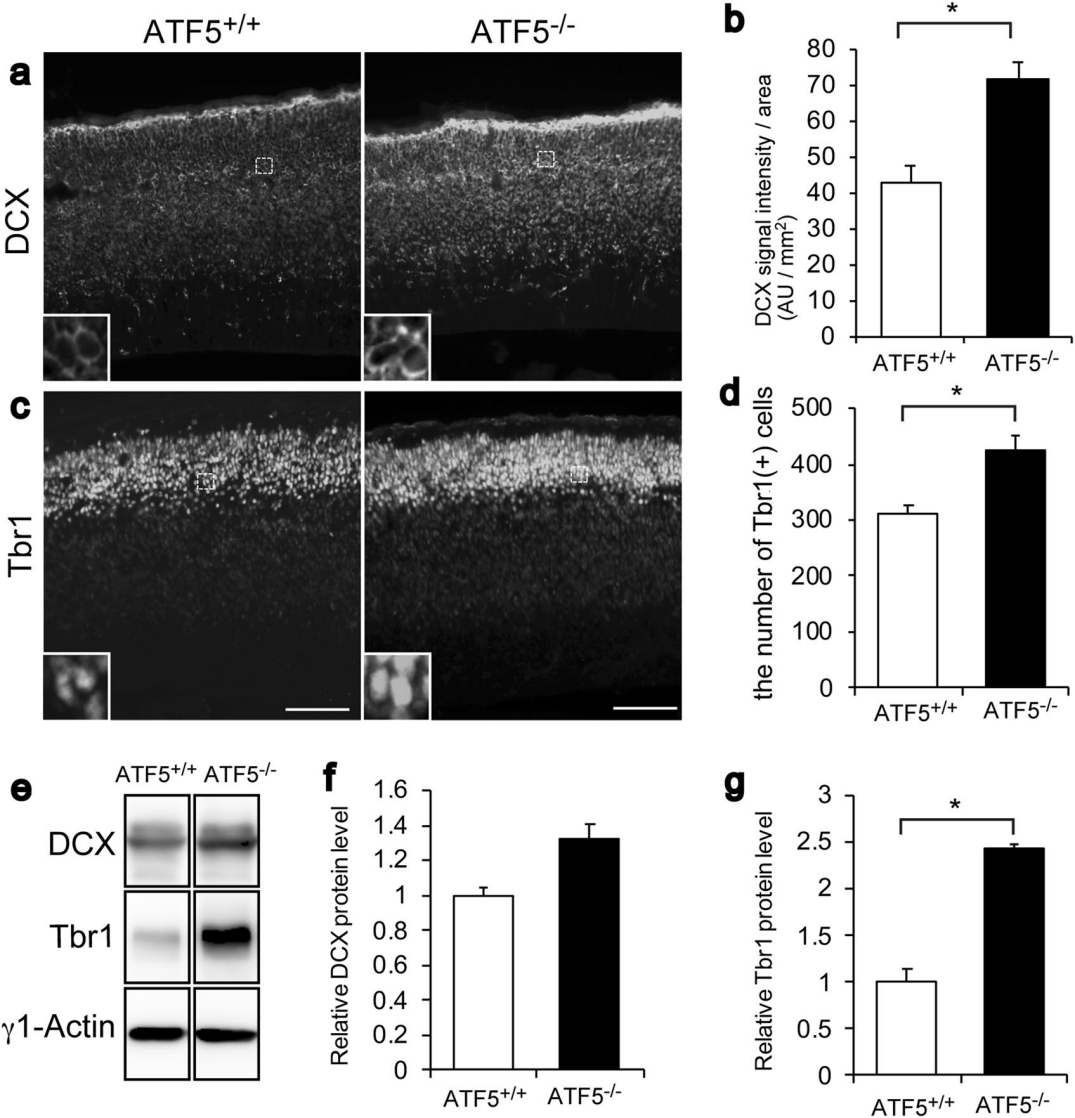
Increased numbers of immature neurons in the developing cortices of ATF5^−/−^ mice. (**a**–**d**) Immunostaining for the immature neuron markers DCX (**a**) and Tbr1 (**c**) in cortical sections on embryonic day 15 (E15). (**b**) Quantification of DCX intensity in images of DCX-immunostained tissues. (**d**) Quantification of the number of Tbr1-positive cells. Each inset shows an enlarged view of the dotted line box. (**e**) Western blotting analysis of DCX and Tbr1 protein levels in cortical lysates from ATF5^+/+^ and ATF5^–/–^ mice on E15. γ1-Actin was used as a loading control. (**f**,**g**) Quantification of the relative band intensities of DCX and Tbr1 normalised to γ1-actin. Data are presented as mean ± S.E.M. (*n* = 3 mice per group in (**a**–**d**), *n* = 5 mice per group in (**e**–**g**), *p < 0.05; Student’s t-test). Scale bars represent 100 µm.

The maintenance of progenitor cells, including radial glial cells and intermediate cells, was reduced in the developing cortex of ATF5^−/−^ mice. Next, we focused on immature neurons by immunostaining for the specific markers doublecortin (DCX) and Tbr1 in cortical sections obtained on E15. We observed increases in DCX protein expression and the number of Tbr1-positive cells in the developing ATF5^−/−^ cortex (Fig. 4a–d). A western blotting analysis demonstrated increased Tbr1 protein levels in lysates of ATF5^−/−^ mouse cortices collected on E15 (Fig. 4e–g). These results suggested that ATF5 deficiency affected immature neuron production during cortical development on E15.

### ATF5 deficiency impaired the localization of certain neurons into upper cortical layers

**Figure 5:**
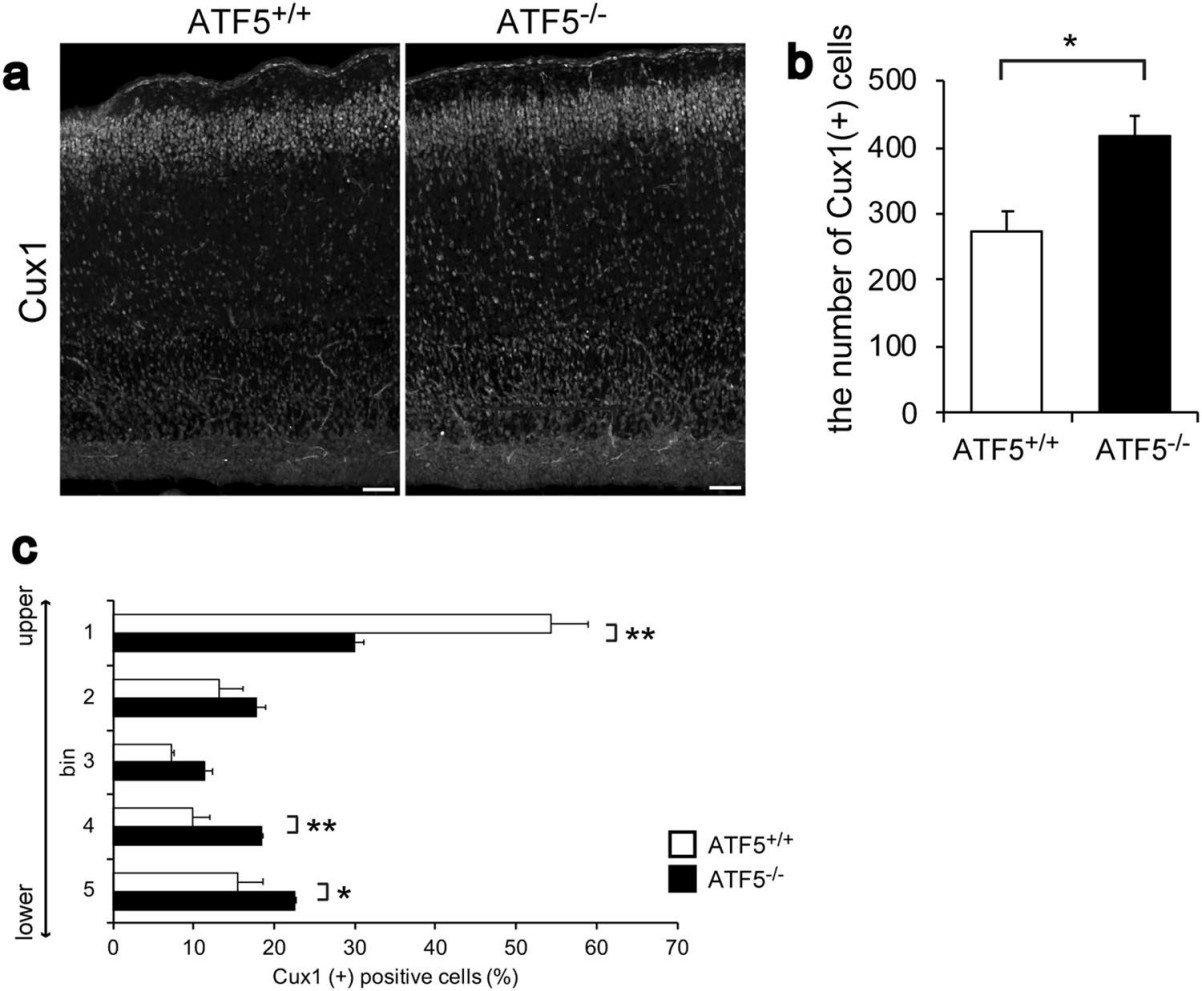
Abnormal number and distribution of upper cortical layer-specific neurons in the ATF5^−/−^ cortex on postnatal day 0 (P0). (**a**,**b**) Immunostaining for Cux1, an upper cortical neuron marker (layers II–VI), in cortical sections on P0. (**b**) Quantification of the number of Cux1-positive cells in the cortex. (**c**) Quantification of the percentage of Cux1-positive cells divided equally into five bins of the cortex. Data are presented as mean ± S.E.M. (*n* = 3 mice per group, *p < 0.05, **p < 0.01; Student’s t-test (**b**), two-way ANOVA followed by Tukey HSD test (**c**)). Scale bars represent 50 µm.

Next, to investigate mature neurons in the cortices of ATF5^−/−^ mice, we performed immunostaining for cortical layer-specific neuronal markers (Cux1 for layer II–IV and Ctip2 for layer V/VI) using postnatal day 0 (P0) mouse cortical sections. In the rodent cortex, radial glial cells generate IPCs on approximately E14. These IPCs migrate and differentiate into mature neurons in the upper cortical layers II–VI. The number of Cux1-positive cells on P0 was higher in the ATF5^−/−^ cortex than in the ATF5^+/+^ cortex (Fig. 5a,b). Quantification of the localization of Cux1-positive cells in each bin revealed an alternative distribution of Cux1-positive cells in the ATF5^−/−^ mouse cortex relative to that in the ATF5^+/+^ mouse cortex (Fig. 5c). In the ATF5^−/−^ mouse cortex, the percentage of Cux1-positive cells in the upper cortical layers was decreased and the percentage of Cux1-positive cells in the lower cortical layers was increased. Immunostaining for Ctip2 revealed that the distribution and number of Ctip2-positive cells were not significantly different between the genotypes (data not shown). These results suggested that ATF5 deficiency affected the localization of upper layer neurons.

### Radial glial cells of ATF5^−/−^ mouse cortex had defective dendrite outgrowth

**Figure 6:**
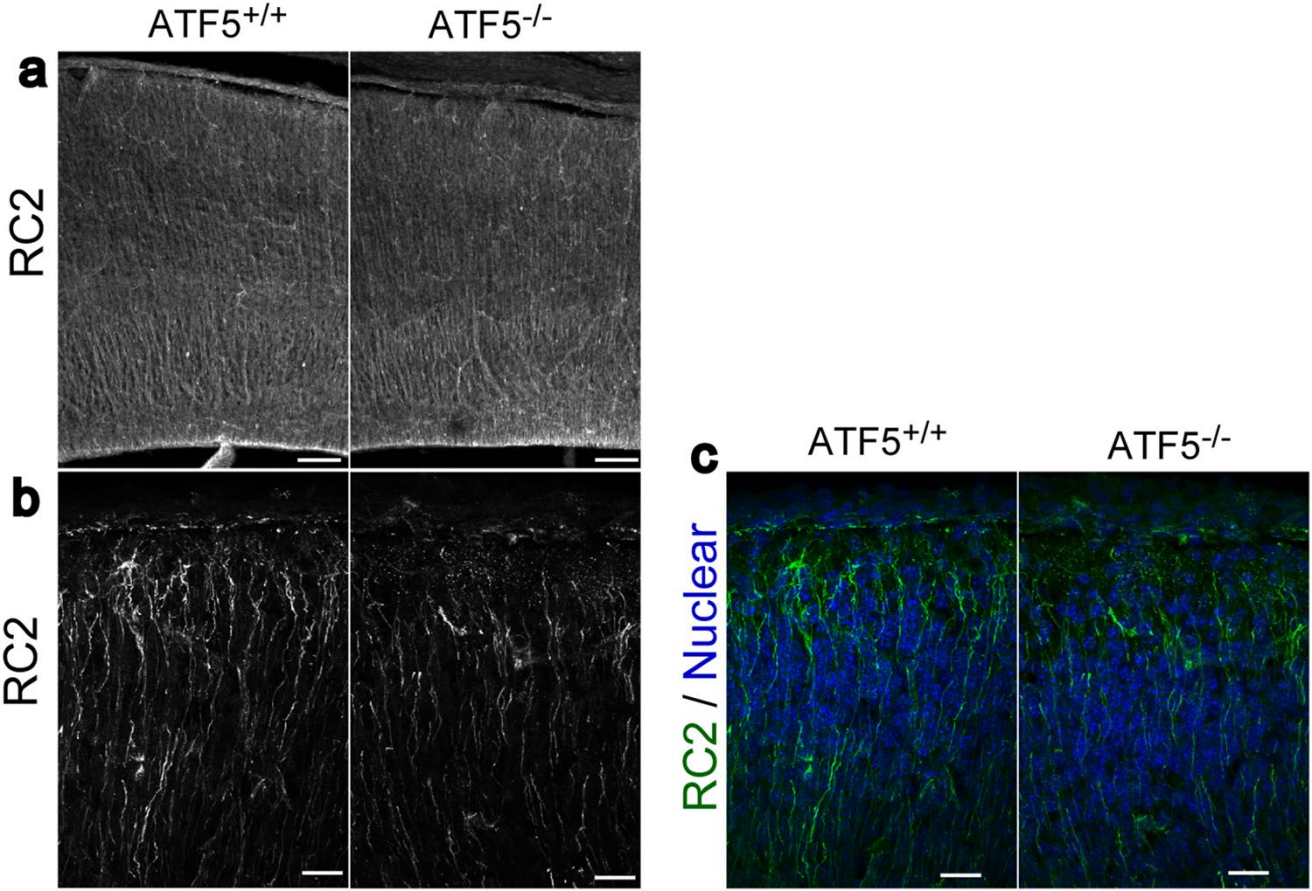
Radial glial process reaching the surface of the cerebral cortex were reduced in the ATF5-deficient mice. (**a**–**c**) Immunostaining of cortical sections with an RC2 antibody on embryonic day 18 (E18). Higher magnification images are shown in (**b**) and (**c**). Scale bars represent 100 µm in (**a**), 20 µm in (**b**) and (**c**).

As shown in Fig. 3, ATF5 deficiency affected the number of radial glial cells. We analysed the radial glial cell morphology, as these cells provide an important scaffolding for neural migration during cortical development. During the late embryo phase (E14–E16 in mice), newly born neurons migrate along scaffolds of radial glial dendrites in a process termed locomotion. We performed immunostaining using an RC2 antibody to investigate the morphology of these radial glial dendrites, which reach the superficial cortex. The dendrites were decreased in the superficial cortices of ATF5^−/−^ mice (Fig. 6a–c), compared to ATF5^+/+^ mice.

### ATF5 deficiency affected neurite outgrowth in culture cells

**Figure 7:**
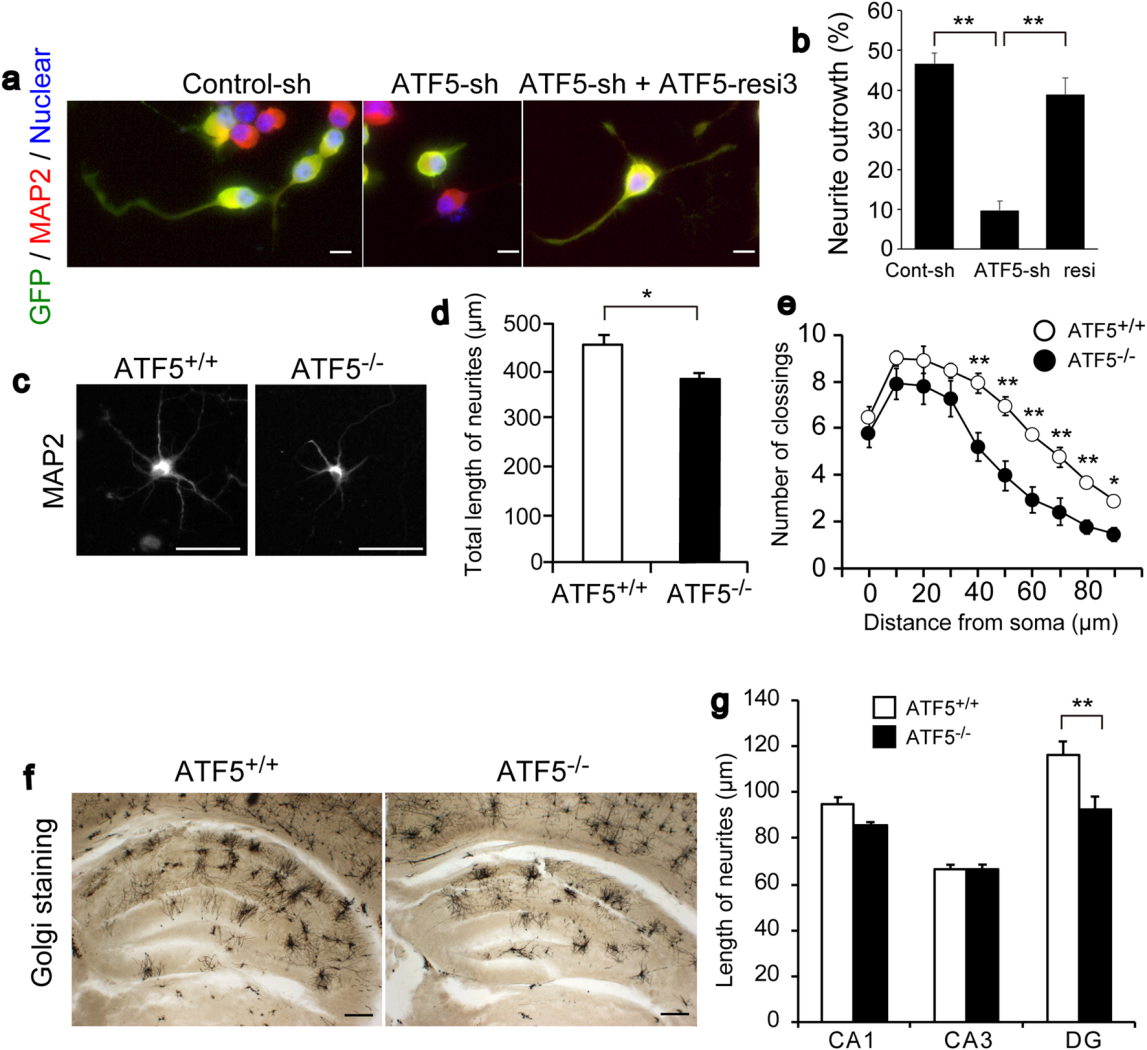
ATF5 deficiency affected dendritic outgrowth in vivo and in vitro. (**a**,**b**) Immunostaining for GFP and the dendritic marker MAP2 in Neuro-2a cells transfected with ATF5-sh or Control-sh with or without ATF5-resi. A GFP expression vector was introduced into Neuro-2a cells. After 1 day of transfection, the medium was replaced with serum-free medium to induce the differentiation of Neuro-2a cells. After 3 days, the Neuro-2a cells were fixed and immunostained. (**b**) Quantification of the percentage of neurite outgrowth in GFP / MAP2 double-positive cells. The rate of neurite outgrowth was determined by the population of neurons with neurites longer than the cell body diameter. (**c**) Immunostaining for MAP2 in hippocampal primary cells isolated from mice on E17 and cultured for 7 days. (**d**) Quantification of the total lengths of neurites. (**e**) Sholl analysis of the MAP2-stained dendrite from the hippocampal primary cells. (**f**,**g**) Golgi-Cox staining for hippocampal neurons in adult mice. (**g**) Quantification of the lengths of the longest dendrites in each hippocampal region. Data are presented as mean ± S.E.M. (*n* = 3 independent experiments (**b**), *n* = 4 independent cell culture experiments per mice group (**d**,**e**), *n* = 4 mice per group (CA1 and DG in (**g**)) and *n* = 6 mice per group (CA3 in (**g**)), *p < 0.05, **p < 0.01; one-way ANOVA followed by Tukey–Kramer test (**b**), Student’s t-test (**d**), two-way ANOVA followed by Tukey–Kramer test (**e**,**g**)). Scale bars represent 10 µm in (**a**), 50 µm in (**c**), and 200 µm in (**f**).

To assess the effect of ATF5 deficiency on neurite outgrowth, we investigated this process by suppressing ATF5 expression in Neuro-2a cells. We constructed a p*Silencer*-based RNAi plasmid (shRNA) against mouse ATF5 (ATF5-sh) and an ATF5 expression construct resistant to this shRNA (ATF5-resi) (Fig. S1a). Endogenous ATF5 mRNA expression was suppressed by the transfection of the ATF5-sh plasmid into Neuro-2a cells (relative to the transfection of control-sh) (Fig. S1b). The expression of FLAG-tagged ATF5 was also suppressed by the transfection of ATF5-sh and was rescued by the co-transfection of FLAG-tagged ATF5-resi with the ATF5-sh plasmid (Fig. S1c). Either ATF5-sh or control-sh was transfected transiently with a GFP expression plasmid into Neuro-2a cells, and the cells were then co-immunostained with anti-GFP and anti-MAP2 antibodies to mark the dendrites. GFP-positive ATF5-knockdown Neuro-2a cells exhibited impaired neurite outgrowth when compared with the controls (Fig. 7a,b). This neurite outgrowth impairment was rescued by co-transfection with ATF5-resi. The total neurite lengths of primary hippocampal cells from ATF5^−/−^ embryos were shorter than those of cells from ATF5^+/^^+^ embryos (Fig. 7c,d). In addition, the complexity of dendrites was determined using Sholl analysis, which measures the number of times dendrites pass through concentric circles localized at various distances from the cell body^24^. Using this analysis, we found that primary hippocampal cells in ATF5^−/−^ mice had higher dendrite complexity than the primary hippocampal cells in ATF5^+/+^ mice (Fig. 7e). We then subjected adult brain sections to Golgi-Cox staining and analysed the dendrite lengths of hippocampal pyramidal neurons (Fig. 7f,g). The dendritic lengths of hippocampal DG (Dentate Gyrus) pyramidal neurons were lower in ATF5^−/−^ mice than in ATF5^+/+^ mice. These results collectively suggested that ATF5 deficiency affected dendritic outgrowth in vivo and in vitro.

### Alternative distribution of upper cortical neurons in adult ATF5^−/−^ mice

**Figure 8:**
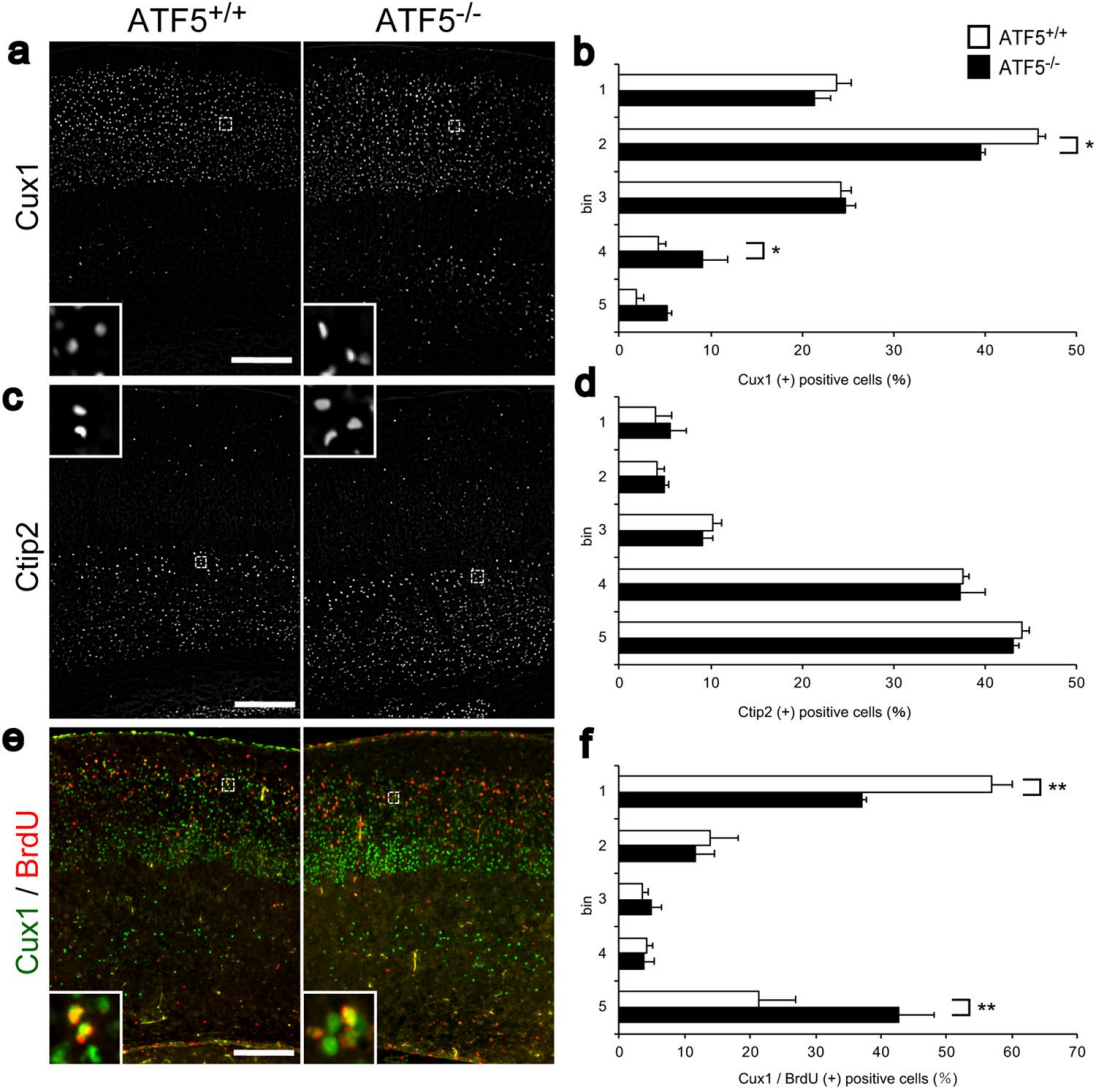
ATF5 deficiency impaired the localization of upper cortical layer neurons in the ATF5^−/−^ cortex. (**a**–**d**) Immunostaining for Cux1, an upper cortical layer neuron marker (layer II–VI) (**a**), and Ctip2, a lower cortical layer neuron marker (layer V/IV) (**c**), in cortical sections on postnatal day 21 (P21). (**e**) Immunostaining for BrdU and Cux1 in P7 cortical sections of offspring from mice that were injected intraperitoneally with BrdU on embryonic day 16 (E16; 200 mg/kg maternal body weight) and dissected on P7. (**b**,**d**,**f**) Quantification of the percentages of Cux1-positive cells (**b**), Ctip2-positive cells (**d**), or Cux1/BrdU double-positive cells (**f**) divided equally into five bins of the cortex. Each inset shows an enlarged view of the dotted line box. Data are presented as mean ± S.E.M. (*n* = 3 mice per group, *p < 0.05, **p < 0.01; two-way ANOVA followed by Tukey HSD test). Scale bars represent 200 µm.

To further examine the role of ATF5 in cortical laminar organization in the adult brain, we investigated the neural distribution of layer-specific neuronal markers (Cux1 for layer lI–IV and Ctip2 for layer V/VI) in ATF5^+/+^ and ATF5^−/−^ mouse cortices. Compared to the ATF5^+/+^ cortex, the ATF5^−/−^ cortex exhibited an altered distribution of Cux1-positive cells on P21 (Fig. 8a,b). Although most Cux1-positive cells were positioned in the upper layer in the ATF5^−/−^ cortex, the percentage of Cux1-positive cells in the lower layer was significantly increased. To explore laminar organization at the same embryonic time point (E16), we administered BrdU on E16 and analysed BrdU-labelled cells for co-immunostaining with Cux1 on P7. The positions of BrdU- and Cux1-double positive cells were altered in the ATF5^−/−^ cortex (Fig. 8e,f). In ATF5^−/−^ mice, the percentage of double-positive cells was significantly increased in the upper layer and decreased in the lower layer. However, the distribution of Ctip2-positive cells was not significantly different between the two genotypes (Fig. 8c,d). These results indicated that ATF5 deficiency affected the neuronal position of layer II–IV neurons and highlighted the involvement of ATF5 in laminar organisation.

## Discussion

Abnormalities in cerebral cortical development underpin several psychiatric and neurodevelopmental disorders^12^. Multiple processes, including neuronal proliferation, migration, differentiation, and functional circuit formation, are tightly regulated by various molecules to ensure precise cortical development. In this study, we demonstrated that ATF5 plays an essential role in embryonic neurogenesis and neural migration during cortical development. ATF5 deficiency resulted in an abnormally thinner cortex and decreased numbers of radial glial cells (Figs. 1, 3). A deficiency in ATF5 also reduced the neurite outgrowth in radial glial cells of the cerebral cortex and culture cells. Collectively, we demonstrated that ATF5 may modulate radial glial cell morphology and survival, which is critical to the ability of these cells to provide a scaffold for neural migration.

ATF5 deficiency resulted in reduced numbers of radial glial cells and IPCs during cortical development (Fig. 3). As shown in Fig. 3, ATF5 deficiency affected the number of Pax6-positive cells, a radial glial cell marker. The number of Tbr2-positive cells, an IPC marker, was also affected. Radial glial cells undergo asymmetrical cell division to generate IPCs and give rise to upper layer neurons during cortical development. These findings are in line with our hypothesis that ATF5 regulates cortical development and maintains radial glial cell survival. The loss of ATF5 function consequent to a dominant-negative ATF5 construct was shown to decrease progenitor cells and increase postmitotic neurons and neurogenesis in cultured telencephalic cells^5^. In this study, ATF5 deficiency promoted immature neuron production during cortical development on E15 (Fig. 4). In the cortex, while most projection neurons arise after a division of the IPCs during cortical development via a process termed ‘indirect neurogenesis’, some of which are generated from apical progenitor cells at early developmental stages via a process termed ‘direct neurogenesis’^15,25,26^. ATF5 deficiency might be thought to contribute to direct neurogenesis or a shift to indirect neurogenesis and to promote the number of immature neurons.

We found that suppression of ATF5 expression inhibits neurite outgrowth in cultured cells. In addition, radial glial dendrites in the superficial cortex were reduced in ATF5^−/−^ mice. This result may be due, at least in part, to a reduced number of radial glial cells. However, these results suggest that ATF5 deficiency affected neurite outgrowth in vivo and in vitro. It is speculated that ATF5 regulates neurite outgrowth. In the future, it will be necessary to investigate the mechanism by which ATF5 is involved in neurite outgrowth. On the other hands, we observed an aberrant distribution of upper layer neurons but not lower layer neurons in the postnatal ATF5^−/−^ mouse cortex. Some Cux1-positive neurons were localized in lower cortical layers (Fig. 8). On E16, most BrdU-labelled newly born neurons did not reach their final positions in the upper cortex (Fig. 8). Projection neurons, which are ultimately localized in the upper cortical layers, are generated by a division of radial glial cells, migrate radially along radial glial processes as a scaffold, and reach their final positions through a migration mode termed ‘locomotion’^14^. ATF5 deficiency affected the morphology of radial glial cells, including the radial dendrites used as a scaffold for locomotion, and therefore at least partly contributed to defects in this migration mode. During cortical development, the centrosome plays an important role in establishing cell polarity^22,27,28^. Particularly, the neuronal centrosome plays important roles in neurogenesis and neural migration. The ATF5 protein is localized in the mother centriole of the centrosome and regulates centrosome function^29^. ATF5 may thus contribute to neuronal migration during cortical development. Future live imaging studies of slice cultures from an ATF5^−/−^ mouse brain will provide more details about migration, including neuronal locomotion.

We previously demonstrated that ATF5^−/−^ mice exhibited abnormal behaviours including hyperactivity in novel environments, increased anxiety-like behaviour, reduced social interaction, and reduced behaviour flexibility^8^. Therefore, we surmise that ATF5^−/−^ mice may provide a useful model for the study of psychiatric disorder pathology, including ASD, anxiety disorder, hyperactivity disorder, and so on. Defects in neurogenesis and the radial migration of cortical neurons were observed in mouse models of neurodevelopmental disorders and psychiatric disorders^12,30–35^ Although the pathogenesis of psychiatric disorders are complex and remain unclear, impaired neuronal migration during cortical development is thought to be a risk factor for psychiatric disorders in humans including ﻿schizophrenia and ASD^36–38^. The abnormal behaviour exhibited by ATF5^−/−^ mice may be at least partly attributable to cortical developmental defects, especially radial migration. Future work should clarify the physiological function of ATF5 in cortical development and elucidate the mechanisms underlying behavioural abnormalities in ATF5^−/−^ mice. Furthermore, it may be important in the future, to generate conditional deficient mice lacking ATF5 in radial glial cells and/or intermediate progenitor cells, and investigate the effects of ATF5 deficiency in cortical development and mouse behaviour analysis. This will be expected to clarify the physiological role of ATF5 in exhibiting abnormal behaviours.

In conclusion, we have demonstrated that ATF5 regulates cortical development. ATF5 deficiency caused defects in cortical development and radial migration. We evaluated survival and morphology and found that ATF5 played an important role in maintaining radial glial cell survival and morphology. Cortical developmental defects in ATF5-deficient mice may partly contribute to abnormal behaviours.

## Methods

### Mice

In this study, homozygous ATF5-deficient (ATF5^−/−^) mice and their littermates wild-type (ATF5^+/+^) mice were used for analysis. This is because our group and other groups have reported that the effects of ATF5 deficiency are not detected in heterozygous ATF5-deficient (ATF5^+/−^) mice^6,20^. ATF5^−/−^ mice and their wild-type (ATF5^+/+^) littermates were generated by mating heterozygous ATF5-deficient (ATF5^+/−^) mice^8,20^. Mice were genotyped via PCR after weaning^20^. These mice have been backcrossed into the C57BL/6N for > 19 generations. The day of vaginal plug detection and the day of birth were designated as embryonic day 0 (E0) and postnatal day 0 (P0), respectively. For all experiments with mice, mice were genotyped before or after use and the sex of embryos and postnatal pups was not determined. All mice were maintained under specific pathogen–free conditions. All procedures involving animals were approved by the Laboratory Animal Care and Committee of the Tokyo university of Pharmacy and Life Sciences, and were performed in accordance with institutional and governmental guidelines. We confirmed that all animal studies were performed in compliance with the ARRIVE guidelines.

### Construction of plasmids

The ATF5 shRNA-targeted sequence in the order of sense, loop (underlined), and antisense was: 5ʹ-GCTAATTGAGGTGTATAAGGCTTCAAGAGAGCCTTATACACCTCAATTAGC-3ʹ. To generate the plasmid encoding ATF5-sh, a pair of primers were annealed, and the product was inserted into the *Bam*HI/*Hin*dIII restriction sites of the p*Silencer* 3.1-H1 neo vector (Thermo Fisher Scientific, Invitrogen, Waltham, MA, USA)^11^. The following primers were used: mATF5sh2F, 5ʹ-GATCCGCTAATTGAGGTGTATAAGGCTTTTCAAGAGAGCCTTATACACCTCAATTAGCTTTTTTA-3ʹ; mATF5sh2R, 5ʹ-AGCTTAAAAAAGCTAATTGAGGTGTATAAGGCTCTCTTGAAGCCTTATACACCTCAATTAGCG-3ʹ. Construction of the control-sh plasmid was described previously^11^. To generate the plasmid encoding the ATF5 mutant resistant to ATF5-sh, we introduced the mutation via site-directed mutagenesis using the mutagenic primers ATF5resi3-R, 5ʹ-GCCTTATAGACTTCGATTAGCAGGTCCTTCACGTAC-3ʹ and ATF5resi3-F, 5ʹ-CTAATCGAAGTCTATAAGGCCCGAAGCCAGAGG-3ʹ. A FLAG-tagged mouse ATF5 expression vector was inserted into the p3xFLAG-CMV-14 vector as a template^39^.

### Immunofluorescence

Immunofluorescence was performed as described previously^20^. In brief, all mice were sacrificed by cervical dislocation. The heads of embryos and neonatal pups (P0) and the dissected brains of mice (> P2) were fixed in 4% paraformaldehyde (PFA) in phosphate-buffered saline (PBS) overnight at 4 °C. The samples were then cryoprotected in 20% sucrose in PBS containing 0.1% (v/v) Tween 20 (PBST) and embedded in Tissue-Tek OCT compound. Coronal and sagittal cryosections (respective thicknesses: 16 and 50 μm) were cut with a cryostat (NX50, Thermo Fisher Scientific). Tissue sections on slides were blocked with 3% bovine serum albumin (BSA) and 0.3% Triton X-100 in PBS for 1 h. In some experiments, a 5% normal donkey serum or 1% blocking reagent solution was used instead of BSA. Antigen retrieval was performed by heating the sections in 10 mM citrate buffer (pH 6.0) using a microwave or Dako Pascal device prior to blocking. After blocking, the specimens were incubated with the primary antibodies shown in Table S1 at 4 °C overnight, washed, and incubated with Alexa Fluor 594- or Alexa Fluor 488-labeled secondary antibodies. Free-floating 50-µm-thick sections were permeabilized with 0.1% Triton X-100 in PBS, blocked with 1% blocking reagent in PBST, and incubated overnight at 4 °C with primary antibodies diluted in blocking solution. After three washes in PBS, the sections were incubated with Alexa Fluor 594- or Alexa Fluor 488-labeled secondary antibodies, and mounted with fluorescent mounting medium (Dako, Glostrup, Denmark). The nuclei were counterstained with Hoechst 33258 (Nacalai Tesque, Kyoto, Japan). Images of immunofluorescence-labelled sections were obtained using a fluorescence microscope (BZ-9000; KEYENCE, Osaka, Japan) or a confocal microscope (FV1000; Olympus, Tokyo, Japan). The primary antibodies are described in the supplementary material.

### Labelling with bromodeoxyuridine (BrdU)

Pregnant mice were injected intraperitoneally with BrdU (200 mg/kg maternal body weight) on E14 or E16 and sacrificed after 24 h or on P7, respectively^17,20,34^. The brains or heads were harvested at the indicated time-points, fixed, and cryosectioned at a thickness of 16 µm as described above. The tissue sections were subjected to antigen retrieval in 10 mM citrate buffer (pH 6.0) using a microwave prior to blocking and stained using an anti-BrdU antibody (details in Table S1).

### Lysate preparation and immunoblotting

To prepare brain lysates, pregnant mice were sacrificed by cervical dislocation, and the cerebral cortices were harvested from the embryo heads. Each cortex was homogenised, and proteins were extracted in RIPA buffer [25 mM Tris–HCl, pH 7.5, 150 mM NaCl, 1% (w/v) NP-40, 1% (w/v) sodium deoxycholate, 0.1% (w/v) SDS] with cOmplete protease inhibitors (Roche Diagnostics, Indianapolis, IN, USA) as described^20^. To prepare cell lysates, the cells were washed with PBS and harvested using RIPA buffer with cOmplete protease inhibitor. Cell debris was removed by centrifugation. The protein concentrations in the lysates were determined using the BCA Protein Assay kit (Thermo Fisher Scientific). Proteins (10 μg per sample) were separated by SDS-PAGE (10% [w/v] polyacrylamide gel) and transferred to a PVDF membrane (Merck Millipore, Billerica, MA, USA). The following primary antibodies and dilutions were used: anti-DCX (dilution, 1:1000; Santa Cruz Biotechnology, Dallas, TX, USA; sc-8066), anti-Tbr1 (1:1000; Abcam, Cambridge, UK; ab31940), and anti-γ1-actin (1:10,000; Wako, Osaka, Japan; 013-24553).

### Cell culture and transfections

Neuro-2a cells were obtained from the JCRB Cell Bank. The cells were grown in Dulbecco’s modified Eagle’s medium (DMEM) supplemented with 10% fetal bovine serum (FBS) at 37 °C and 5% CO_2_ in a humidified chamber. Neuro-2a cells were transiently transfected with ATF5-sh or Control-sh with or without ATF5-resi using the FuGENE 6 Transfection Reagent (Promega, Madison, WI, USA) according to the manufacturer’s instructions. For differentiation, at 24 h after transfection, Neuro-2a cells were cultured in serum-free medium (DMEM without FBS) for 2 or 3 d. For immunoblotting, the cells were harvested 2 d after transfection.

For hippocampal primary neuron isolation, pregnant mice were sacrificed on E17 by cervical dislocation, and the hippocampi were isolated into Hanks’ Balanced Salt solution (HBSS) buffer (Wako). The hippocampi were digested using 0.1% Trypsin in HBSS for 20 min at 37 °C. FBS was added to stop trypsin digestion. After digestion, DNA was disrupted using 0.05% DNase I and 10 mM MgSO_4_ in HBSS for 3 min, and the hippocampal neurons were dissociated. The neurons were washed and resuspended in Neurobasal media (Thermo Fisher Scientific, Invitrogen) supplemented with B-27 (Thermo Fisher Scientific, Invitrogen) and L-glutamate, plated on glass coverslips (Matsunami, Osaka, Japan), coated with 0.1 mg/mL poly-L-lysin (Sigma), and cultured for 7 days.

### Immunocytochemistry and cell morphology analysis

Neuro-2a cells and hippocampal primary neurons were plated on glass coverslips coated with poly-L-lysine for the cell morphology analysis. The cells were fixed in 4% paraformaldehyde in PBS or ice-cold methanol for 20 min. Next, the cells were blocked with PBS containing 3% BSA and 0.2% Triton X-100. The samples were then incubated with primary antibodies diluted in PBS containing 1% BSA and 0.2% Triton X-100 at 4 °C overnight or at room temperature for 2 h. After washing in PBS, the samples were incubated with Alexa Fluor 594- or Alexa Fluor 488-labelled secondary antibodies and mounted with fluorescent mounting medium. The cell nuclei were stained with Hoechst 33258. Images were obtained with a fluorescence microscope (BZ-9000; KEYENCE) or a confocal microscope (FV1000; Olympus). The primary antibodies used were anti-GFP (dilution, 1:300, rabbit; MBL; 598) and anti-MAP2 (1:100, mouse; Sigma; M4403).

### Quantification of neurite outgrowth and complexity using the Sholl analysis

In the neurite outgrowth analysis of Neuro-2a cells subjected to ATF5 knockdown (ATF5-sh) and rescue with the ATF5-resi plasmid, photographs of random fields were obtained 3 d after serum withdrawal for differentiation using a fluorescence microscope (BZ-9000; KEYENCE). GFP-positive cells bearing neurites were analysed using NIH software (ImageJ, Bethesda, MD, USA). A minimum of 75 GFP-positive cells were measured per condition. The percentage of neurite outgrowth was calculated by counting the GFP-positive cells bearing neurites that were longer than the diameter of the cell body^40–42^. In a neurite outgrowth analysis of hippocampal primary culture neurons isolated from ATF5^−/−^ and ATF5^+/+^ mice, 10 neurons per independent cell culture of each mice group were randomly selected. The lengths of all dendrites from the cell body were measured, and the sum of the dendrite lengths and number of neurites from the cell body were calculated. In neurite outgrowth analysis of Golgi-stained hippocampal neurons, the lengths of longer dendrites in each area of the hippocampus were measured using ImageJ. The complexity of dendrites was analysed using the Sholl analysis^24,43^ using the Image J software. We performed Sholl analysis with a 10 µm ring interval starting from the soma. For Sholl analysis, five hippocampal primary cells per independent culture of each mice group was selected.

### Golgi-Cox staining

Golgi-Cox staining was performed using the FD Rapid GolgiStain Kit (FD Neuro Technologies, Ellicott City, MD) according to the manufacturer’s instructions, as previously described^44^. Briefly, the brains of adult mice were removed quickly, rinsed in distilled water, and incubated in a mixture of solutions A and B (1:1) from the kit for approximately 3 days at room temperature in the dark. Subsequently, the brains were transferred into solution C from the kit and stored for 3 days at 4 °C in the dark. The brains were then frozen on powdered dry ice and stored at −80 °C until cryosectioned. The brain tissues were sectioned into 100-μm-thick slices using a cryostat (NX50, Thermo Fisher Scientific). The sections were placed on gelatinised slides, rinsed in distilled water, and immersed in a mixture of solutions D and E from the kit and distilled water and incubated in the dark, followed by another rinse in distilled water. The slides were subsequently dehydrated in a series of ethanol solutions (50, 75, 95, 100%) for 4 min each, covered, and stored in the dark until completely dry. Hippocampal neurons were analysed using a microscope (BZ-9000; KEYENCE).

### Quantification and statistical analysis

ImageJ software was used to quantify the cells and cortical thicknesses. For the latter measurements, the thicknesses of the cortical sections with labelled nuclei were measured over the overall thickness of the cortex (from the pial to ventricular surface). Cell counts were analysed as previously described^22^. To obtain cell counts in the cerebral cortex, all cells in a 450-µm-wide area from the ventricular to the pial surface were quantified using ImageJ software. For counts of PHH3-positive cells, cells in a 450-µm-wide columnar area of the ventricular surface and extra-ventricular surface were quantified.

Statistical analysis was conducted using JMP14 (SAS Institute Inc., Cary, NC, USA) and Statplus: mac (AnalystSoft Inc., version v7). Data were analysed by one-way or two-way ANOVA (analysis of variance) followed by Tukey HSD (honestly significant difference) test or Tukey–Kramer test, and Student’s t-test. All quantitative data are given as mean ± S.E.M. The significance level of the statistical difference was set at *p* ≤ 0.05 (*p < 0.05, **p < 0.01).

## Supplementary Information

Supplementary information.
