# Supplementary material for: ATF5 deficiency causes abnormal cortical development
Source: Sci Rep. 2021 Mar 31;11:7295. doi: 10.1038/s41598-021-86442-5 (PMC8012588; doi:10.1038/s41598-021-86442-5)

Supplementary information

Title

ATF5 deficiency causes abnormal cortical development

Authors

Mariko Umemura, Yasuyuki Kaneko, Ryoko Tanabe, Yuji Takahashi

Supplementary information

1. Supplementary Figure S1: Construction of a pSilencer-based RNAi plasmid (shRNA) against mouse ATF5 (ATF5-sh) and an ATF5 expression construct resistant to this shRNA (ATF5-resi).
2. Supplementary Table S1: Antibody used in immunohistochemistry of this study.

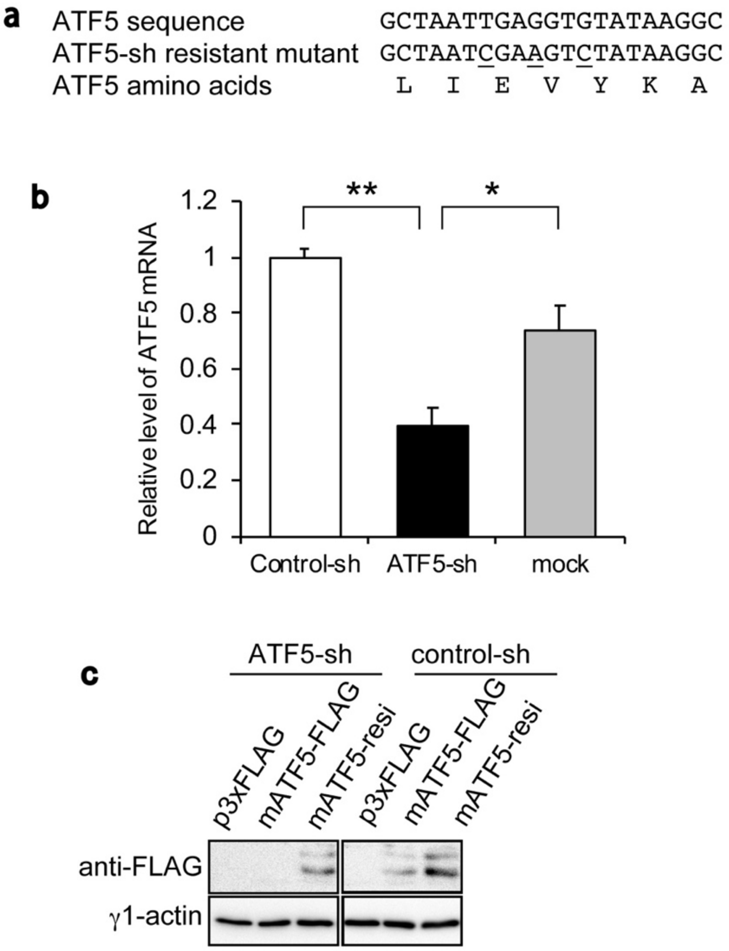

Figure S1. Construction of a pSilencer-based RNAi plasmid (shRNA) against mouse ATF5 (ATF5-sh) and an ATF5 expression construct resistant to this shRNA (ATF5-resi). (A) The DNA sequence of ATF5-sh and ATF5-sh resistant mutant (ATF5-resi) and the amino acid sequence of ATF5. (B) Quantification analysis of endogenous ATF5 mRNA level of using real time PCR. (C) Western blotting analysis of the FLAG-tagged mouse ATF5 (mATF5-FLAG) expression levels. Neuro-2a cells were transiently transfected with p3xFLAG (empty vector), p3xFLAG-mATF5 (mATF5-FLAG), and p3xFLAG-mATF5-resi (mATF5-resi) when co-transfected with or without ATF5-sh or Control-sh. After 2days transfected, lysate was isolated and analyzed by western blotting analysis using FLAG antibody. The expression of FLAG-tagged ATF5 was suppressed by the transfection of ATF5-sh and was rescued by the co-transfection of FLAG-tagged ATF5-resi with the ATF5-sh plasmid. γ1-Actin was used as a loading control. Data are presented as mean ± S.E.M. ($n = 3$, $*p < 0.05$, $**p < 0.01$).

Table S1. Antibody used in immunohistochemistry of this study

Antibody to	Source	Species	Dilution	Catalog No.
BrdU	Bio-Rad	rat	1:200	MCA2060
Ctip2	Abcam	rat	1:500	ab18465
Cux1	Santa Cruz	rabbit	1:50	sc-13024
DCX	Santa Cruz	goat	1:250	sc-8066
Ki67	Leica Biosystems	rabbit	1:400	NCL-Ki67p
Ki67	BD Biosciences	mouse	1:50	550609
PHH3 (Ser10)	Millipore	rabbit	1:100	06-570
Pax6	BioLegend	rabbit	1:500	901301
RC2	DSHB	mouse	1:200	RC2-a
Sox2	Abcam	rabbit	1:200	ab97959
Tbr2	Abcam	rabbit	1:200	ab23345
Tbr2 (EOMES)	Invitrogen	rat	1:500	14-4875-80
Tbr1	Abcam	rabbit	1:500	ab31940

Supplementary information: Full blot Images of western blotting in the Manuscript

Title

ATF5 deficiency causes abnormal cortical development

Authors

Mariko Umemura, Yasuyuki Kaneko, Ryoko Tanabe, Yuji Takahashi

Supplementary information

1. Full blot Image of western blotting in Figure 4(e)
2. Full blot Image of western blotting in Supplementary Figure S1 (c)

Description

Squares with dot lines, the location of images used in the main figures.

Figure 4(e) (Full blot image of WB in Figure 4e)

DCX

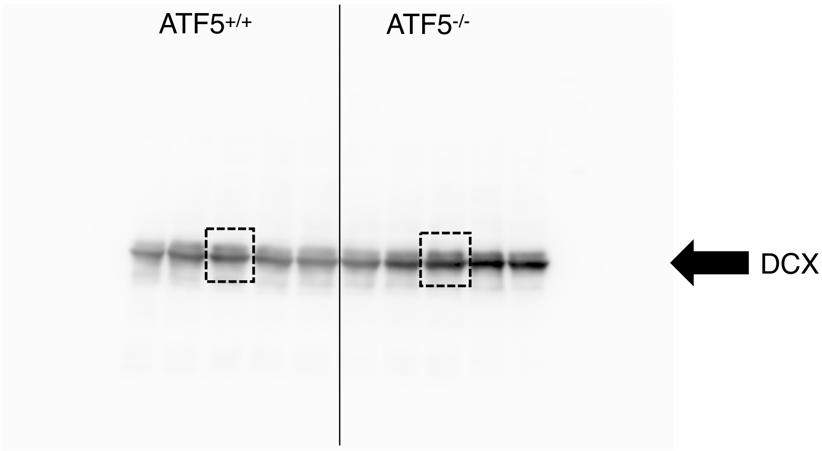

Tbr1

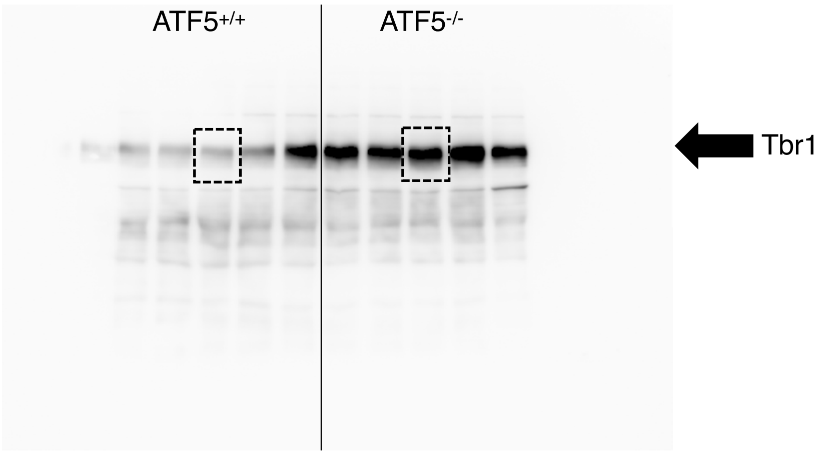

γ1-Actin

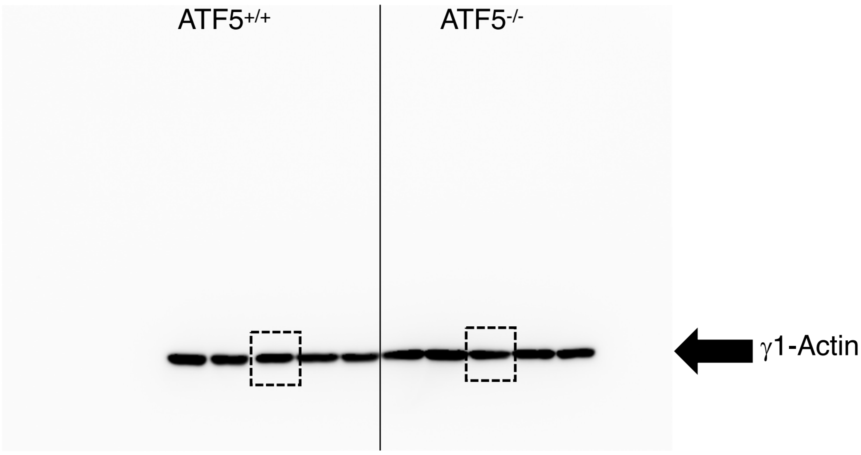

Supplementary Figure S1(c) (Full blot image of WB in Figure S1c)

FLAG

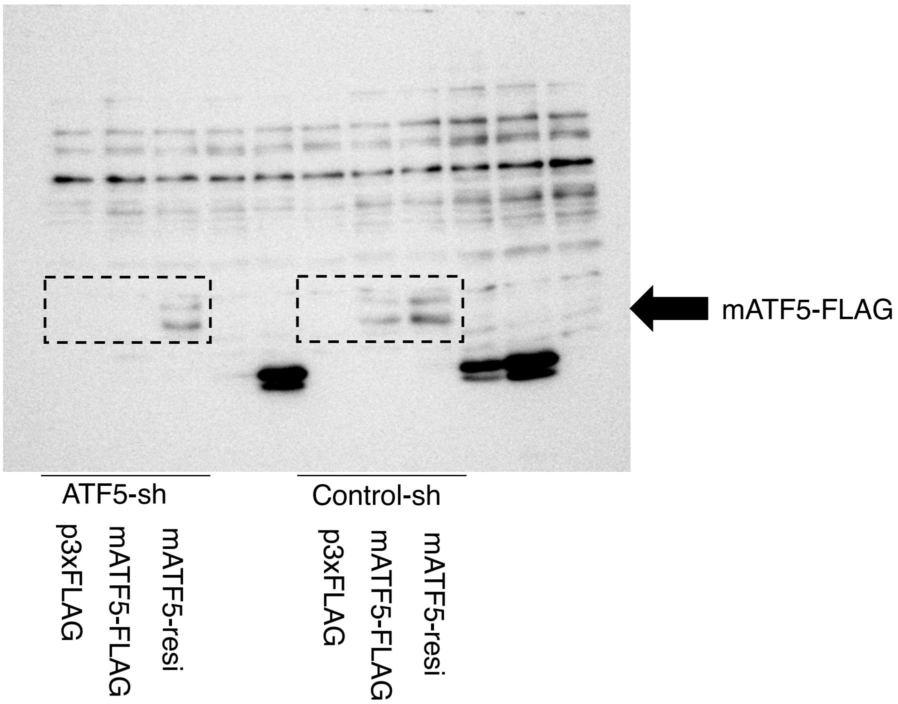

γ1-Actin

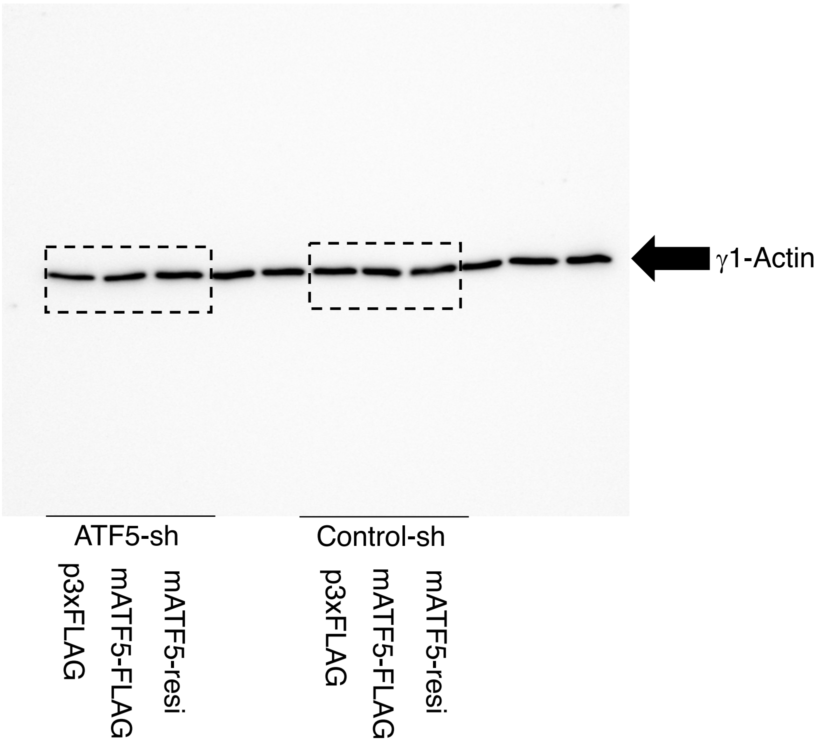

Supplement: Supplementary file 1 — Supplementary information. [file 41598_2021_86442_MOESM1_ESM.pdf]
